# Do Dietary Supplements Improve Perceived Health Well-Being? Evidence from Korea

**DOI:** 10.3390/ijerph18031306

**Published:** 2021-02-01

**Authors:** Donghoon Kim, Inbae Ji, John N. Ng’ombe, Kwideok Han, Jeffrey Vitale

**Affiliations:** 1 Department of Food Industrial Management, Dongguk University, 30 Pildong-ro 1-gil Jung-gu, Seoul 04620, Korea; yohan6575@naver.com; 2 Department of Agricultural Economics and Extension, University of Zambia, Lusaka 10101, Zambia; ngombe@okstate.edu; 3 Department of Institutional Research and Analytics, Oklahoma State University, 203 PIO Building, Stillwater, OK 74078, USA; kwideok.han@okstate.edu; 4 Department of Agricultural Economics, Oklahoma State University, 418 Ag Hall, Stillwater, OK 74078, USA; jeffrey.vitale@okstate.edu

**Keywords:** average intake effects, dietary supplements, health well-being, propensity score matching

## Abstract

This study analyzes the self-reported intake of dietary supplements (DS) and their effects on perceived health well-being from a survey with 1210 adult respondents in Korea. To account for selectivity bias from observable confounders, we use a propensity score matching (PSM) model. Our findings show that demographics, health concerns, family history of disease, frequency of hospital visits, and regular exercise are positively associated with intake of DS among consumers. Results from PSM show that the intake of DS leads to significant improvements in perceived health well-being among DS takers relative to DS non-takers regardless of gender, urban residence, having self-reported diseases or not. The paper concludes with implications for policies that promote intake of DS in Korea.

## 1. Introduction

People’s quest for a healthy lifestyle is an important aspect of consumer choice and demand theory. Beginning with Grossman’s [1] economic framework, which models an individual’s health as an initial stock that declines over time, economists have investigated causal linkages between short and long-term dietary choices and health outcomes [2,3]. Individuals make investments in their health through proper dieting, exercising, and other lifestyle choices to maintain their health at existing levels. Other less appropriate choices such as smoking, substance abuse, inactivity, and poor dietary habits lead to a more rapid decline in an individual’s health [4].

Dietary guidelines suggested by most health councils and agencies stress the importance of consuming the recommended types of foods and drinks required for a healthy lifestyle [5]. This typically includes fresh fruits and vegetables, and a balanced intake of meats, dairy products, and grains [6]. Since lifestyles, costs, and other circumstances often make it difficult for individuals to maintain an adequate diet, dietary supplements (DSs) are often taken to meet and/or enhance nutritional requirements [7,8,9,10]. In a more general setting, DSs are taken to promote overall health [11]. Over the past decades, DSs have become an important component of the food industry in many countries. In the U.S., about half of consumers regularly take at least one DS with total sales reaching $25.1 billion in recent years [12,13]. While not intended to prevent, diagnose, treat, or cure diseases, DSs provide consumers with a range of products to address a variety of health concerns. These health concerns include vitamin deficiencies, heart ailments, energy boost, weight control, sleep aid, colds management, and general health of bones, joints, and eyes [11,12,14,15]. Dietary supplements encompass a wide range of active ingredients, including vitamins (A, C, B_6_, B_12_, D, and E), calcium, omega-3 fatty acids, protein supplements, botanicals, fiber, potassium, magnesium, folic acid, and iron [11].

One of the most significant trends in healthcare is the positive income effect on investments in individual health. As national and per capita incomes rise, studies have confirmed a corresponding increase in investments in health and economic growth [16,17]. In Korea, the country which has experienced substantial economic growth over the past few decades, consumer investment in healthcare has also increased dramatically. Increased attention to personal health has also been associated with single-person households, which have become more prevalent. Emerging out of this desire has been a growth in the consumption of DSs. Some Korean consumers refer to DSs as “healthy functional foods”. This is expected in part due to the fact that DSs are fairly new to Korean consumers. However, typical consumers of DSs in Korea are aware of these products because DSs are legally classified and noticeable with certification marks. Contrary to most DSs, functional foods are easier to be differentiated from DSs because the former are part of the customary diet with specific disease prevention attributes [18]. For example, the Food and Nutrition Board of the Institute of Medicine defines a functional food as any food or food ingredient that may provide a health benefit exceeding traditional nutrients that it contains [19]. Basically, DSs are responsible for general health benefits while functional foods may provide specific health benefits since they are considered part of the diet [20].

While fairly new to Korean consumers, DS products have experienced rapid growth and have gained nationwide popularity. According to the Korea Health Supplements Association [21], 73.6% of Korean households bought at least one DS product in 2018 and demand levels are expected to grow continuously over the next decade. Total sales of DS products in 2018 were valued at ₩2.5 trillion (i.e., about $2.27 billion) which accounted for 0.14% of GDP, 0.52% of manufactural industry GDP, and 2.66% of the overall value of the food industry. Total sales (including domestic and imports) reached ₩3.1 trillion in 2018. Sales over the past five years for the industry have grown by an average rate of 11.2% annually. The export value of DS products was ₩12.6 billion in 2018 representing a 17.1% annual growth rate. The total imports were valued at ₩673 billion representing a 10.3% annual growth rate since 2014. Thus, the DS industry is expected to continue with rapid growth in the near future and therefore increase its role and value to the national economy.

A dietary supplement is defined as the food that is manufactured or processed with the ingredients and components that have useful function/structure for the human body (Korean Government’s “Dietary Supplement Act (DSA) of 2002” [22]. Conditions and stipulations set forth by the DSA of 2002 are similar in nature to the landmark 1994 Dietary Supplement Health and Education Act (DSHEA) legislation passed in the United States. In Korea, DS must be approved by the Ministry of Food and Drug Safety (MFDS), and should be separated from prescriptive drugs, conventional and functional foods. DS products are labeled as DS based on the DS Act in Korea.

Since factors that affect an individual’s health are diverse, quantifying a single factor’s effect on health is a difficult and complicated process. Since the endogeneity of the variables affecting health well-being cannot be controlled accurately, it could potentially create bias by conflating the effect of factors having a similar effect on health. Such bias could lead to erroneous policy implications and conclusions. The goal of this study is to analyze the determinants of DS intake and their effects on health well-being among Korean consumers. Specifically, this paper analyzes the determinants of intake of DS in Korea. We also determine the effects that consumption of DS have on consumers’ perceived health well-being in Korea. To remedy any potential endogeneity from self-selection into taking DS products by consumers, this study uses propensity score matching (PSM) [23] to quantify the intake effects of DS products on perceived health well-being. The PSM remains a useful econometric tool for analyzing causal effects in observational studies. It has therefore been used in analyzing various policy issues including healthcare, agricultural policy, and employment. Our paper aims to provide the first rigorous empirical evidence on the DS intake effects on perceived health well-being in Korea.

## 2. Literature Review

In general, DS products are taken as a prophylactic to prevent illness and promote improved health rather than as a prescribed drug [13]. Consumers expect that DSs will improve their health, but it is difficult to quantify their effect. In the case of prescription drugs, clinical trials are used to determine the efficacy of alternative treatment regimens on human (and animal) subjects. Typically, DSs have clinical trials performed to obtain admission into government regulated frameworks, leaving substantial gaps for trials after they have reached the marketplace.

Previous research on DS products has studied various aspects of the consumer choice problem for DS, including the personal characteristics of DS takers, motivational factors for taking DS, the perception of the effectiveness of DS, and knowledge of DS. Often DSs are taken to address long term healthcare needs and prevention, such as cancer, heart disease, liver health, and others that are difficult to test in the immediate sense [11]. Studies in England have confirmed the positive effects of a healthy lifestyle, age, and gender (female) on DS use [24,25]. DS users in the US cite improving (45%) or maintaining (33%) overall health as the most common reasons for taking DSs rather than targeting individual concerns, according to the National Health and Nutrition Examination (NHANES) survey conducted from 2007 to 2010 [11]. Other frequently mentioned reasons cited in the NHANES survey were typically more directed at a single function, including improving bone health (25%), lowering cholesterol (15.1%), weight loss (2.6%), supplementing diets (22.0%), preventing arthritis (12.4%), and energy boost (10.8%) [11]. DS takers were allowed in the survey to cite multiple reasons hence percentages cited in the text could exceed 100% in total. Many studies have identified athletes and individuals involved in personal training regimes to take DS for performance enhancement and energy boosting.

Many studies analyzing DSs identify a typical DS taker as one with the following attributes: older, more active, and prone to exercise, in better self-reported physical condition with lower body mass index (BMI), less likely to smoke and drink alcohol, and more formally educated compared to non-takers [7,11,21,22]. Studies from other countries have found similar characterizations of DS users, though differences have also been identified. In Sweden, effects of age, self-reported health, exercise, and body weight have been reported as being consistent with US findings [23]. Swedish women were found, however, to be more likely DS takers than men and neither smoking nor education had any influence on use. Anders and Schroeter [13] studied the relationship between DS intake and obesity outcomes (BMI) in the United States. They found a negative relationship between DS use and BMI, suggesting a positive effect on health for takers interested in weight loss.

Similar issues have been addressed in the Korean literature on DS including consumer perceptions, motivation, and attitudes towards various attributes related to DS purchases [26,27,28,29,30,31,32]. Marketing issues related to the promotion of DS products have been addressed by studies in the literature. These include development of functional ingredients and their promotion and labeling in DS [33,34,35,36,37,38,39,40,41,42]. Although the DS industry in Korea has experienced rapid growth, there is a void in research assessing the intake effects of DS on consumers’ health. This study contributes to literature by being the first to examine the effects of DS products on consumers’ perception of health well-being in Korea.

## 3. Materials and Methods

### 3.1. Theoretical Framework

It is non-trivial to quantify the intake effect of DS because factors that affect an individual’s health are diverse. To estimate the cause and effect with a special variable, the most important step for these experiments is random selection assignment (random selection for experiment is to assign samples randomly between treatment group and comparison group, so, any treatment receives an equal chance between two groups) between treatment and comparison samples. Since social science studies differ from natural science studies, non-random selection is the norm. To solve non-random selection bias and therefore to closely make the sample random, quasi-experimental methods have been introduced [43]. Several econometric methods have been used to examine causal effects of the treatment variables. In our case, the simplest method would be to use ordinary least squares (OLS) with a dummy variable of the DS intake variable in the outcome equation, while assuming DS intake as being strictly exogenous. However, DS intake may be endogenous, for it could be influenced by intake desire or motivation of the participating individual that may take DSs based on expected health benefits. Thus, OLS estimates under these conditions would be biased. A possible solution to solving the endogeneity problem is using instrumental variables. However, the challenge with this method is the identification of a strong and suitable instrumental variable. To control for the challenges of heterogeneity, possible endogeneity and self-selectivity of DS intake by individuals, this study uses PSM techniques to estimate intake effects of DSs on health well-being among consumers.

### 3.2. Propensity Score Matching (PSM)

Following Rosenbaum and Rubin [44], the average treatment effect (ATE) can be calculated as

(1) $$ATE_{i}=Y_{i1}-Y_{i0}$$

where *Y_i1_* is the perceived health well-being of if consumer *i* took DS product,$Y_{i0}$ is the perceived health status if consumer *i* did not take DS product. However, both outcomes would not be observed simultaneously in non-experimental studies. What is usually observed is

(2) $$Y_{i}=D_{i}Y_{1i}+(1-D_{i})Y_{0i}$$

where *D_i_* = 1 or 0.

According to Becerril and Abdulai [45], letting *P_r_* to denote the likelihood of observing an individual with *D_i_ =* 1, ATE could be specified as

(3) $$\begin{array}{l} ATE_{i}=P_{r}.[E(Y_{1i}|D_{i}=1)-E(Y_{0i}|D_{i}=1]+\\ \left(1-P_{r}).[E(Y_{0i}|=0)-E(Y_{0i}|D_{i}=0)\right] \end{array}$$

Equation (3) implies that the causal effect for the sample is the weighted average effect for adopting on the treatment and control groups. Each group is weighted by its relative frequency. The counterfactuals $E(Y_{1i}|D_{i}=0)$ and $E(Y_{0i}|D_{i}=1)$ cannot be estimated given that the available data provide no information on the counterfactual situation [46]. In such situations, Blundell and Costa-Dias [47] propose the method of statistical matching when estimating the direct effect of an innovation or technology from the difference in results across individuals. As earlier mentioned, this study further uses PSM as an econometric framework.

PSM is a semiparametric approach that involves constructing a statistical comparison group by modeling the probability of participating in the program based on observed characteristics that are unaffected by the program [44]. Basically, the PSM model matches the treatment and control groups based on the predicted probability to adopt a superior activity [44,46,48]. The model is attractive because it compares the treatment group’s observed results with those of the comparison group [48].

The propensity score is defined as the conditional probability of receiving the treatment given the pretreatment variables [44]. In this study, the DS takers are matched based on the propensity score, to DS non-takers. A propensity score is defined as

*P*(*X*) = *Pr* (*D_i_* = 1|*X)* = *E (D_i_ |X)) → P(X) = F{h(X)}* (4)

where *F*{.} can be either normal or logistic cumulative distribution function and *X* is a vector of observed covariates. Estimation of causal effects using PSM relies on the conditional independence and overlap assumptions. Takahashi and Barrett [49] suggest that conditional independence assumes that there is statistical independence between the potential outcome of interest and probability of adoption conditional on observed covariates. The overlap condition is stated as

(5) $$0\;<\;Pr\;(D_{i}\;=\;1|X_{i}\;=\;x)\;<\;1\to \forall X_{i}$$

The overlap assumption implies that the support of the conditional distribution of *X_i_* given *D_i_* = 0 overlaps completely with that of the conditional distribution of *X**_i_*** given *D_i_* = 1. Thus, the average treatment effect on the treated (ATT) is defined in the common support region only. Following Becerril and Abdulai [45] and Lu et al. [50], upon computation of the propensity scores, ATT can be computed as follows:

(6) $$\begin{array}{ll} ATT_{i} & =E(Y_{1i}-Y_{0i}|D_{i}=1)\\ & =E[E(Y_{1i}-Y_{0i}|D_{i}=1,P(X)]\\ & =E[E(Y_{1i}|D_{i}=1,P(X))-E(Y_{0i}|D_{i}=0,P(X))|D_{i}=1]\; \end{array}$$

Several matching methods exist in the literature though the most frequently used are the nearest-neighbor matching (NNM) and kernel matching (KM). NNM consists of matching treatment and control groups that have closest propensity scores. KM matches the treatment groups with a weighted mean of all control groups using weights that are inversely proportional to the difference in magnitude of the propensity scores of the former and latter. Following Becerril and Abdulai [45], the ATT is estimated upon the calculation of the difference of each matched pair’s unit. For robust results, it is recommended that quality of matching is checked. Rosenbaum and Rubin [44] and Caliendo and Kopeinig [51] suggest the standardized mean difference between treatment and control groups in which they recommend that a standard difference exceeding 20% is too large, an indicator of failure of the matching process. Furthermore, a comparison of the pseudo R-squared and *p*-values for likelihood ratio tests following probit regression analysis before and after matching are also recommended.

### 3.3. Data Sources and Variable Description

Data were collected from 1210 adults as part of a nationwide survey in Korea (those below 19 years old, children and adolescents were excluded from our survey). To make the survey representative, the survey sample was drawn in proportion to Korean demographic characteristics such as region, age, and gender. The internet survey method was used. To verify the survey’s accuracy, we first conducted a pilot survey. Since consumers could be confused between functional food and DSs, we informed consumers about how the law defines DSs and how DSs are labeled before surveying. Consumers were informed that:

*“According to the Korean Government’s Dietary Supplement Act, DSs are manufactured or processed using raw materials or ingredients that have functions useful for the human body. DSs are distinguished from medicines (drugs), functional foods, and general foods. DS products have the phrase ‘Dietary Supplement’ or ‘certification mark’ on their packing. DS examples include vitamins, glucosamine, lutein, chlorella, amino acids, red ginseng, etc.”.*

A DS taker in this study was a person that said yes to have taken at least one of the following products in the previous year: vitamins, lutein, chlorella, amino acids, red ginseng, calcium, iron, *Echinacea*, garlic, fish oils, probiotics, and glucosamine. The survey included respondents’ demographics, pattern of DS consumption and intake effect, self-reported health well-being (compared to a year ago), if they had any diseases in 2018, respondents’ medical treatment, and current diet. We classified each individual as treatment and non-treatment (control) group based on whether they took DSs at least once in 2018.

Descriptive statistics indicate that 86% of respondents took DS during 2018. The respondents’ health concern was 3.96 based on the 5-point Likert scale, and disease experience in 2018 was 55.7%. About 82.5% of DS takers were concerned about their health compared to 50.6% DS non-takers that were concerned about their health. Diseases experience (individual diseases were surveyed using 32 main diseases codes based on the Korean welfare panel survey) was higher among DS takers (at 56.5%) than DS non-takers (at 50.6%). Family history of disease was 40.4%. The BMI was calculated from self-reported height and weight. BMI is an individual’s weight divided by the square of its height and based on this index, individual is classified by underweight (below 18.5), health (18.5–22.9), light overweight (23–24.9), overweight BMI (25–29.9), and obesity (above 30). Based on the calculated BMI, 57.1% would be considered obese.

The average number of hospital visits by DS non-takers was 5.3 times in 2018 while DS takers visited the hospital 5.7 times in 2018. Average medical expenses were ₩394,000 for DS takers while they were ₩216,000 for DS non-takers. Respondents who had more hospital visits might be more interested in DSs because of the belief that they enhance health. However, it is noteworthy to mention that DSs are not substitutes for established medicines, as the DSs would not treat their ailments. The result of average respondents’ lifestyle shows that drinking alcohol was high (61.1%) and smoking was low (19.3%). About 42% of respondents answered their diet was regular, but only 21.6% indicated that they had nutrient balance in their diet. Thus, the result shows that respondents’ eating behaviors were not healthy. In the questionnaire, respondents were asked to choose a score that represented their health well-being compared to the previous year before taking DSs. The health well-being was defined as a perceived value between 0 and 10 with 0 being the worst while 10 being the best health well-being score. From Table 1 the health well-being compared to a year ago was 5.55. The health well-being summary results for the overall sample, DS takers, and non-takers compared to a year ago are shown in Table 1. On average, the health well-being of DS takers had a score of 4.54 while for DS non-takers, it was 5.71.

### 3.4. Empirical Model

In our analysis, exogenous variables are socioeconomic characteristics (gender, age, residence, marriage, and the number of family), self-reported health status (health concern, health care, diseases, family history of diseases, and BMI), lifestyle, and diet (alcohols, smoking, regular exercise, and sleeping hours). The treatment variable is the intake of DS product while the outcome variable is the perceived health well-being, as defined in Table 2. To estimate individual’s propensity score, we estimated a logit regression. We then predicted the probability for intake of DSs and used it as a propensity score, estimated the common support region and found a range of probability, and confirmed the level of overlapping between treatment and control group. To estimate the propensity score, the logit model was specified as:

(7) $$\begin{array}{ll} \ln\;\left(intake_{i}\right) & =\beta_{0}+\beta_{1}gender_{i}+\beta_{2}age_{i}+\beta_{3}area_{i}+\beta_{4}married_{i}+\\ & \beta_{5}member_{i}+\beta_{6}edu_{i}+\beta_{7}inc_{i}+\beta_{8}concern_{i}+\beta_{9}disease_{i}+\\ & \beta_{10}family_{i}+\beta_{11}bmi_{i}+\beta_{12}hospital_{i}+\beta_{13}medical_{i}+\\ & \beta_{14}drink_{i}+\beta_{15}smoke_{i}+\beta_{16}exer_{i}+\beta_{17}sleep_{i}+\beta_{18}habit_{i}+\\ & \beta_{19}nutrition_{i}+\varepsilon_{i} \end{array}$$

**Figure 1:**
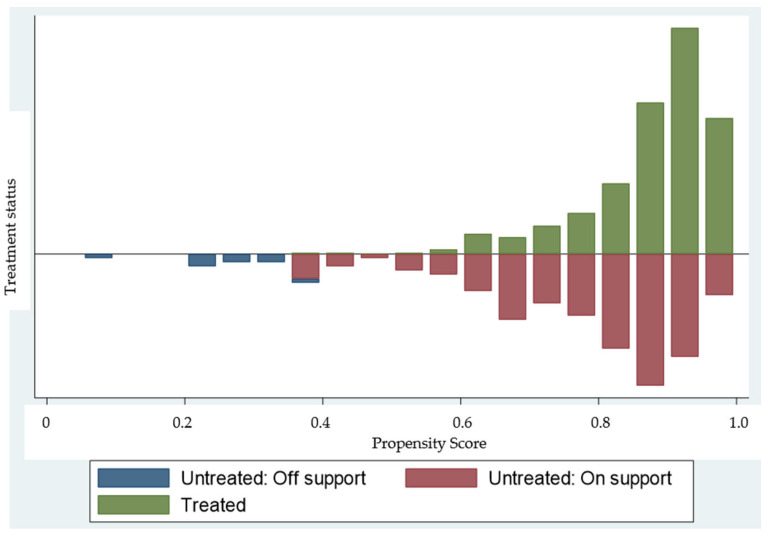
Distributions of the estimated propensity scores between treated and control groups.

After confirming the estimates of propensity score and common support region, we matched groups using NNM and KM (Figure 1).

To check the quality of matching, we conducted a balancing test for exogenous variables before and after matching. We examined the reduction rate of bias using standardized bias and then compared the average bias before and after matching between groups. If conditional independence assumption holds, small values of standardized bias indicates a good matching result since the difference between groups after matching based on the propensity score would be smaller. We also checked whether bias was reduced after matching through by the statistics presented in Table A1 and Table A2 in the Appendix A. Finally, we estimated the ATT of the intake effect of DS takers based on an individual’s characteristics.

## 4. Results

### 4.1. Determinants of Intake of DS Products

Our estimated parameter estimates of the logit model of DS intake are presented in Table 3. The estimated logit model’s McFadden pseudo-R^2^ of 0.153 and correctly predicted 99.3% and 84.7% of DS takers and non-takers, respectively. The logit regression results show that variables that significantly affected the decision to take DSs among consumers include demographics such as gender and marriage status of consumers, health-related factors such as health concerns and family history of diseases, healthcare related factors such as hospital visits, and lifestyle dietary factors. Specifically, female respondents were more likely to take DS than their male counterparts, we found that married consumers were more likely to take DS products than single individuals.

Our results suggest that consumers with health concerns and whose families have history of diseases are more likely to take DS products. Frequent hospital visits are associated with increased likelihood of taking DS products. In terms of lifestyle and diet related factors, only respondents that regularly exercise had a higher probability to take DSs.

### 4.2. The DS Intake Effects on Perceived Health Well-Being

The estimated propensity score from the logit model (based on NNM and KM) ranged from 0.4107 to 0.9999. The total 17 of samples were located in outside of common support region as indicated in (Table 4). So, the total sample was reduced to 1193 to estimate ATT.

Under assumption of independence, we conducted the balance test for exogenous variables (covariates) using nearest neighbor and kernel matching to verify whether matching was performed appropriately. We examined the difference between groups before and after matching, the determinants of both nearest neighbor and kernel matching were reduced since the characteristics between two groups became similar after matching compared to before matching, average and median bias that indicate standardized bias were reduced after matching. Thus, the matching was well performed. To compare whether the means of covariates of two groups were similar after matching, we conducted a *t*-test for each exogenous variable by matching methods. We then analyzed each standardized bias and its reduction of exogenous variables.

The results show that the biases of most of exogenous variables were reduced, as reported in the Appendix A Table A1 and Table A2. We rejected the hypothesis that two groups’ average values of covariates are not different, indicating that matching performance was successful. In the NNM framework, all exogenous variables except for the diseases, alcohols, and normal sleeping hours had reduced bias, while in the KM framework, all of variables with the exceptions of BMI and normal sleeping hours had reduced bias. The variables that were not reduced bias were small average values so it would not affect average treatment effects (ATEs).

We turned our attention to the intake effects of DSs on individual’s perceived health well-being under PSM. We estimated ATTs using the NNM and KM algorithms and results are shown in Table 5. The results indicate DS intake resulted into an increase in perceived health well-being among all DS takers by 10.85–11.76%. Among the male respondents, taking DSs led to increased perceived health well-being by about 11.13–12.21%. For females, our PSM results show that taking DS products led to increased health well-being by about 10.2–10.7%. For respondents from urban areas, DS intake increased self-rated health well-being of DS takers by about 11.6–12.7%. We also considered the causal effects of DS intake among those who had diseases and without. Our results show that DS takers in the sample that had diseases had their perceived well-being increase by about 14.3–14.8% while DS takers who did not have diseases improved by about 4.95–5.20%.

## 5. Discussion and Conclusions

This study analyzed the self-reported intake of dietary supplements (DSs) and their effects on perceived health well-being in Korea. We used a propensity score matching (PSM) framework to account for the potential endogeneity from self-selection problems from observable characteristics among DS taking consumers and to assure credible results. A high number of respondents reported taking DSs, suggesting that taking DS use has been popularized in Korea. Gender and marital status of respondents are significant predictors of the decision to take DSs among the respondents. Female consumers are more likely to take DSs than males. Women are physiologically more likely to be deficient of iron and vitamins [52,53], which may drive them to consume more DSs consistent with previous results [52,54]. With respect to marital status, we found that married consumers are more likely to consume DSs, consistent with previous research [55].

Consumers with health concerns or whose families have a history of diseases are more likely to consume DS because taking DSs enhances the person’s perception of health well-being. While DSs are not medicines, their expected health benefits may be more attractive to people that have health related concerns whether current or based on their family history. These findings are consistent with those reported by Conner et al. [53], where most DS takers strongly believe that taking more DSs help them to be healthier. Thus, those with health concerns or family history of diseases may take DSs to avoid a disease. Frequent hospital visits are associated with a higher probability of taking DS. People with health-related problems are more likely to take DSs to enhance their health well-being [52,54]. Regarding lifestyle and diet related factors, this study found that respondents that exercise regularly are more likely to take DSs and is consistent with some DSs recommended to improve athletic performance [56]. The logit model estimates showed that females, married consumers, health-related factors such as health concerns and family history of diseases, frequent hospital visits play a crucial role at affecting DS intake among consumers. These results are consistent with previous literature (e.g., [53,54,55]). Intake effect results show that taking DSs improves consumers’ perceived health well-being compared to a year ago. We found significant positive effects of taking DSs for both genders, urban and rural residents, and DS takers with and without diseases for those that took DS products. Our results recommend those taking DSs to continue as they the significant potential to, on average, improve their self-rated health well-being.

There were some limitations of this study. First, health well-being was self-reported and measured using scaled scores. Additionally, these scores reported whether the individual’s health had improved from the previous year. This may capture whether the health well-being had improved, but not actually capture the true health well-being of an individual. For future research, we suggest that it should use actual experimental trials to improve results reported in this study. However, the perception of health well-being is an important factor for individuals to measure their own health linked to achieving life satisfaction [57,58]. Thus, findings from this study provide insights for measuring consumers’ perception of the influence of DSs on perceived health well-being. Most importantly, our findings highlight the importance of taking into account of such attributes as gender differences, residence type, and having self-reported diseases or not when evaluating public perceptions of DS effects on health well-being. Due to the popularity of DSs with Korean consumers, government support will still be needed to regulate the DS industry in terms of consumer safety. Another limitation of this study is that the sample of the non-treatment group was smaller than that from the treatment group. Thus, it could have happened not to match efficiently. However, the balancing tests for majority of the covariates were satisfied as indicated in the Table A1 and Table A2 in Appendix A.

## Figures and Tables

**Table 1:** A year comparison of health well-being.

Classification		Get Worse		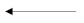		No Change			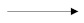		Get Better		Average
		0	1	2	3	4	5	6	7	8	9	10	
Taker	Frequency (%)	3.0	4.0	12.0	32.0	99.0	394.0	216.0	146.0	95.0	19.0	20.0	5.71
		0.3	0.4	1.2	3.1	9.5	37.9	20.8	14.0	9.1	1.8	1.9	
Non-taker	Frequency (%)	7.0	3.0	6.0	16.0	29.0	86.0	10.0	6.0	5.0	–	2.0	4.54
		4.1	1.8	3.5	9.4	17.1	50.6	5.9	3.5	2.9	–	1.2	
Total	Frequency (%)	10.0	7.0	18.0	48.0	128.0	480.0	226.0	152.0	100.0	19.0	22.0	5.55
		0.8	0.6	1.5	4.0	10.6	39.7	18.7	12.6	8.3	1.6	1.8	

**Table 2:** Descriptive statistics (*N* = 1210).

Variable		Mean	Std. Dev.	Min.	Max.
Demographics	Gender (Male = 1)	0.5074	0.5002	0	1
	Age	42.9678	13.4896	19	78
	Residence (Urban = 1)	0.9116	0.2840	0	1
	Marriage (Married = 1)	0.6360	0.4828	0	1
	Household size	3.0537	1.2132	1	9
	Education	2.8620	0.6060	1	4
	Monthly average household income	4.3446	2.3282	1	9
Health State	Health concern	3.9579	0.7178	1	5
	Diseases (Yes = 1)	0.5570	0.4969	0	1
	Family history of diseases (Yes = 1)	0.4041	0.4909	0	1
	BMI (Healthy = 1)	0.4289	0.4951	0	1
Healthcare	Hospital visits	5.3074	8.0096	0	130
	Medical expense	36.8645	69.4631	0	600
Lifestyle and Diet	Alcohols (Yes = 1)	0.6107	0.4878	0	1
	Smoking (Yes = 1)	0.1926	0.3945	0	1
	Regular exercise (Yes = 1)	0.4388	0.4965	0	1
	Normal sleeping hours (Yes = 1)	0.5397	0.4986	0	1
	Regular eating pattern	3.1760	0.9237	1	5
	Nutrient balance diet	2.9430	0.7593	1	5
Treatment	Took supplementary dieter at least once for a year 2018.	0.8595	0.3476	0	1
Outcome	Health well-being (0 to 10)	5.5455	1.5940	0	10

**Table 3:** Logistic regression results for taking dietary supplements.

Variable Name		Coefficients	Standard Error
Dependent variable: Intake of dietary supplements			
Explanatory variables			
Demographics	Gender (Male = 1)	−0.710 ***	0.209
	Age	−0.040	0.050
	Age Squared	0.000	0.001
	Residence (Urban = 1)	0.338	0.298
	Marriage (Married = 1)	0.680 **	0.264
	Household size	−0.130	0.082
	Education	0.053	0.164
	Monthly average household income	0.068	0.044
Health State	Health concern	1.006 ***	0.133
	Diseases (Yes = 1)	−0.241	0.203
	Family history of diseases (Yes = 1)	0.402 **	0.202
	BMI (Healthy = 1)	0.089	0.190
Healthcare	Hospital visits	0.057 **	0.026
	Medical expense	0.002	0.002
Lifestyle and Diet	Alcohols (Yes = 1)	−0.015	0.198
	Smoking (Yes = 1)	0.123	0.243
	Regular exercise (Yes = 1)	0.322 *	0.190
	Normal sleeping hours (Yes = 1)	−0.154	0.185
	Regular eating pattern	0.145	0.124
	Nutrient balance diet	−0.133	0.154
Constant		−1.766	1.159
Log Likelihood		−415.574	
Pseudo $R^{2}$		0.153	

*Notes*: ***, **, * indicate 1%, 5%, 10% at the significant level respectively.

**Table 4:** Range of common supports between dietary supplements taker and non-taker.

Classification	Number of Sample			Range	
	Total	On Support	Off Support	Min	Max
Taker	1040	1040	0	0.4107	0.9999
Non-taker	170	153	17		
Total	1210	1193	17		

**Table 5:** Average treatment effects of intake of dietary supplements on the perceived health well-being.

	NN Matching		Kernel Matching	
Dependent Variable	Difference	T-Stat	Difference	T-Stat
Intake DS Overall Effect	1.1760 ***	6.19	1.0854 ***	6.24
Intake DS for Males	1.1212 ***	4.28	1.1130 ***	4.69
Intake DS for Females	1.0242 ***	4.71	1.0764 ***	5.29
Intake DS for Urban Residents	1.2708 ***	6.34	1.1588 ***	6.30
Intake DS for Rural Respondents	0.3057	0.59	0.0322	0.06
Intake DS for Respondents With Diseases	1.4783 ***	5.25	1.4299 ***	5.40
Intake DS for Respondents Without Diseases	0.4950 **	2.23	0.5199 **	2.60

*Notes*: ***, ** indicate statistical significance at 1% and 5% significance levels, respectively.

## Data Availability

Data used for this study are available upon request from authors.

## Appendix A

**Table A1:** Balance test results for covariates before and after matching (N–N matching).

Variable	Before/After Matching	Average		% Reduction		*T*-test	
		Taker	Non-Taker	%bias	$\left|bias\right|$	*t*	$p>\left|t\right|$
Gender	Before	0.487	0.635	−30.3	93.2	−3.61	0.000
	After	0.508	0.518	−2.1		−0.45	0.653
Age	Before	43.397	40.341	22.8	85.0	2.75	0.006
	After	43.313	42.854	3.4		0.75	0.452
Residence	Before	0.918	0.871	15.5	60.2	2.03	0.042
	After	0.918	0.937	−6.2		−1.63	0.103
Marriage	Before	0.651	0.506	29.6	96.2	3.65	0.000
	After	0.644	0.639	1.1		0.25	0.800
Household size	Before	3.044	3.112	−5.5	5.2	−0.67	0.501
	After	3.046	2.981	5.2		1.22	0.224
Education	Before	2.875	2.782	15.0	13.2	1.85	0.065
	After	2.875	2.794	13.0		2.94	0.003
Monthly average household income	Before	4.416	3.906	22.3	17.4	2.66	0.008
	After	4.372	3.950	18.5		4.18	0.000
Health concern	Before	4.042	3.441	77.2	91.2	10.58	0.000
	After	4.007	4.060	−6.8		−1.76	0.079
Diseases	Before	0.565	0.506	11.9	−2.2	1.45	0.148
	After	0.550	0.610	−12.2		−2.74	0.006
Family history of diseases	Before	0.424	0.282	29.9	90.2	3.50	0.000
	After	0.409	0.395	2.9		0.63	0.527
BMI	Before	0.433	0.406	5.4	26.2	0.65	0.513
	After	0.429	0.409	4.0		0.89	0.372
Hospital visits	Before	5.659	3.159	38.0	85.2	3.79	0.000
	After	4.708	5.078	−5.6		−1.59	0.113
Medical expenses	Before	39.355	21.626	30.0	66.4	3.10	0.002
	After	32.397	26.444	10.1		2.66	0.008
Alcohols	Before	0.607	0.635	−5.9	−11.0	−0.71	0.479
	After	0.618	0.650	−6.5		−1.47	0.143
Smoking	Before	0.188	0.218	−7.2	51.5	−0.89	0.371
	After	0.196	0.181	3.5		0.80	0.422
Regular exercise	Before	0.459	0.318	29.2	79.2	3.45	0.001
	After	0.455	0.425	6.1		1.31	0.189
Normal sleeping hour	Before	0.538	0.547	−1.7	−433.4	−0.21	0.835
	After	0.543	0.590	−9.2		−2.06	0.040
Regular eating pattern	Before	3.205	3.000	22.7	90.8	2.69	0.007
	After	3.200	3.181	2.1		0.46	0.643
Nutrient balance diet	Before	2.956	2.865	12.0	28.1	1.45	0.147
	After	2.963	2.897	8.6		1.91	0.056

**Table A2:** Balance test results for covariates before and after matching (kernel matching).

Variable	Before/After Matching	Average		% Reduction		T-test	
		Taker	Non-Taker	%bias	$\left|bias\right|$	*t*	$p>\left|t\right|$
Gender	Before	0.487	0.635	−30.3	77.5	−3.61	0.000
	After	0.508	0.542	−6.8		−1.49	0.136
Age	Before	43.397	40.341	22.8	81.5	2.75	0.006
	After	43.313	42.749	4.2		0.93	0.353
Residence	Before	0.918	0.871	15.5	78.6	2.03	0.042
	After	0.918	0.928	−3.3		−0.85	0.395
Marriage	Before	0.651	0.506	29.6	97.7	3.65	0.000
	After	0.644	0.648	−0.7		−0.15	0.877
Household size	Before	3.044	3.112	−5.5	95.9	−0.67	0.501
	After	3.0456	3.043	0.2		0.05	0.959
Education	Before	2.875	2.782	15.0	53.0	1.85	0.065
	After	2.875	2.831	7.0		1.58	0.115
Monthly average household income	Before	4.416	3.906	22.3	27.4	2.66	0.008
	After	4.372	4.001	16.2		3.71	0.000
Health concern	Before	4.042	3.441	77.2	96.7	10.58	0.000
	After	4.007	4.027	−2.5		−0.67	0.503
Diseases	Before	0.565	0.506	11.9	30.6	1.45	0.148
	After	0.550	0.591	−8.3		−1.86	0.063
Family history of diseases	Before	0.424	0.282	29.9	78.2	3.50	0.000
	After	0.409	0.378	6.5		1.40	0.160
BMI	Before	0.433	0.406	5.4	−32.0	0.65	0.513
	After	0.429	0.394	7.2		1.60	0.110
Hospital visits	Before	5.659	3.159	38.0	98.9	3.79	0.000
	After	4.708	4.682	0.4		0.12	0.907
Medical expenses	Before	39.355	21.626	30.0	64.0	3.10	0.002
	After	32.397	26.007	10.8		2.85	0.004
Alcohols	Before	0.607	0.635	−5.9	43.2	−0.71	0.479
	After	0.618	0.634	−3.3		−0.75	0.455
Smoking	Before	0.188	0.218	−7.2	81.9	−0.89	0.371
	After	0.196	0.201	−1.3		−0.29	0.769
Regular exercise	Before	0.459	0.318	29.2	86.8	3.45	0.001
	After	0.455	0.436	3.8		0.83	0.406
Normal sleeping hour	Before	0.538	0.547	−1.7	−240.7	−0.21	0.835
	After	0.543	0.573	−5.9		−1.31	0.190
Regular eating pattern	Before	3.205	3.000	22.7	92.0	2.69	0.007
	After	3.200	3.216	−1.8		−0.40	0.687
Nutrient balance diet	Before	2.956	2.865	12.0	81.2	1.45	0.147
	After	2.963	2.946	2.3		0.50	0.614
